# Investigating various metal contacts for p-type delafossite α-CuGaO_2_ to fabricate ultraviolet photodetector

**DOI:** 10.1038/s41598-023-35458-0

**Published:** 2023-05-22

**Authors:** Masoud Abrari, Majid Ghanaatshoar, Shahab Sharifi Malvajerdi, Saeb Gholamhosseini, Alireza Hosseini, Haiding Sun, Seyed Majid Mohseni

**Affiliations:** 1 Laser and Plasma Research Institute, Shahid Beheshti University, Tehran, 1983969411 Iran; 2 School of Microelectronics, University of Science and Technology of China, Hefei, 230026 Anhui China; 3 Department of Physics, Shahid Beheshti University, Tehran, 1983969411 Iran

**Keywords:** Materials science, Electronic devices, Electronics, photonics and device physics, Electronic properties and materials, Semiconductors, Electrical and electronic engineering

## Abstract

Delafossite semiconductors have attracted substantial attention in the field of electro-optics owing to their unique properties and availability of p-type materials that are applicable for solar cells, photocatalysts, photodetectors (PDs) and p-type transparent conductive oxides (TCOs). The CuGaO_2_ (CGO), as one of the most promising p-type delafossite materials, has appealing electrical and optical properties. In this work, we are able to synthesize CGO with different phases by adopting solid-state reaction route using sputtering followed by heat treatment at different temperatures. By examining the structural properties of CGO thin films, we found that the pure delafossite phase appears at the annealing temperature of 900 °C. While at lower temperatures, delafossite phase can be observed, but along with spinel phase. Furthermore, their structural and physical characterizations indicate an improvement of material-quality at temperatures higher than 600 °C. Thereafter, we fabricated a CGO-based ultraviolet-PD (UV-PD) with a metal–semiconductor-metal (MSM) configuration which exhibits a remarkable performance compared to the other CGO-based UV-PDs and have also investigated the effect of metal contacts on the device performance. We demonstrate that UV-PD with the employment of Cu as the electrical contact shows a Schottky behavior with a responsivity of 29 mA/W with a short response time of 1.8 and 5.9 s for rise and decay times, respectively. In contrast, the UV-PD with Ag electrode has shown an improved responsivity of about 85 mA/W with a slower rise/decay time of 12.2/12.8 s. Our work sheds light on the development of p-type delafossite semiconductor for possible optoelectronics application of the future.

## Introduction

Nowadays, CuGaO_2_ (CGO) has widespread application in electro-optical devices due to its substantial optical and electronic properties^1,2^. Delafossite CGO with a 3.6 eV bandgap and its significant conductivity can promise remarkable applications in the ultraviolet (UV) spectrum range. In addition, CGO is an intrinsic p-type semiconductor, which has great importance compared with other transparent conductive oxides (TCOs), such as ZnO, CdO, SnO_2_, In_2_O_3_:Sn, or In_2_O_3_:Mo which are typically n-type semiconductors^3^. So far, p-type TCOs including Cu_2_O, NiO, VO_2_ are the most popular materials for study. The emerging delafossite CGO with a high transmittance of 80% in visible region as well as its tunable hole concentration up to about 10^21^ cm^-3^ has shown its promise as p-type TCOs^4,5^. Moreover, various studies show that the emerging CGO material can be widely used in dye-sensitized solar cells (DSSCs)^6^, photocatalysts^7,8^, p-n junctions^9^, transparent thin film transistors (TTFT)^10^, hole transport layer (HTL) for perovskite solar cells^11,12^, and photodetectors^13^. Additionally, by excellent lattice matching with Ga_2_O_3_ and ZnO, this material can also be promising for fabrication of all-oxide p-n junctions for various optoelectronic and electronic applications^8,14^.

**Figure 1:**
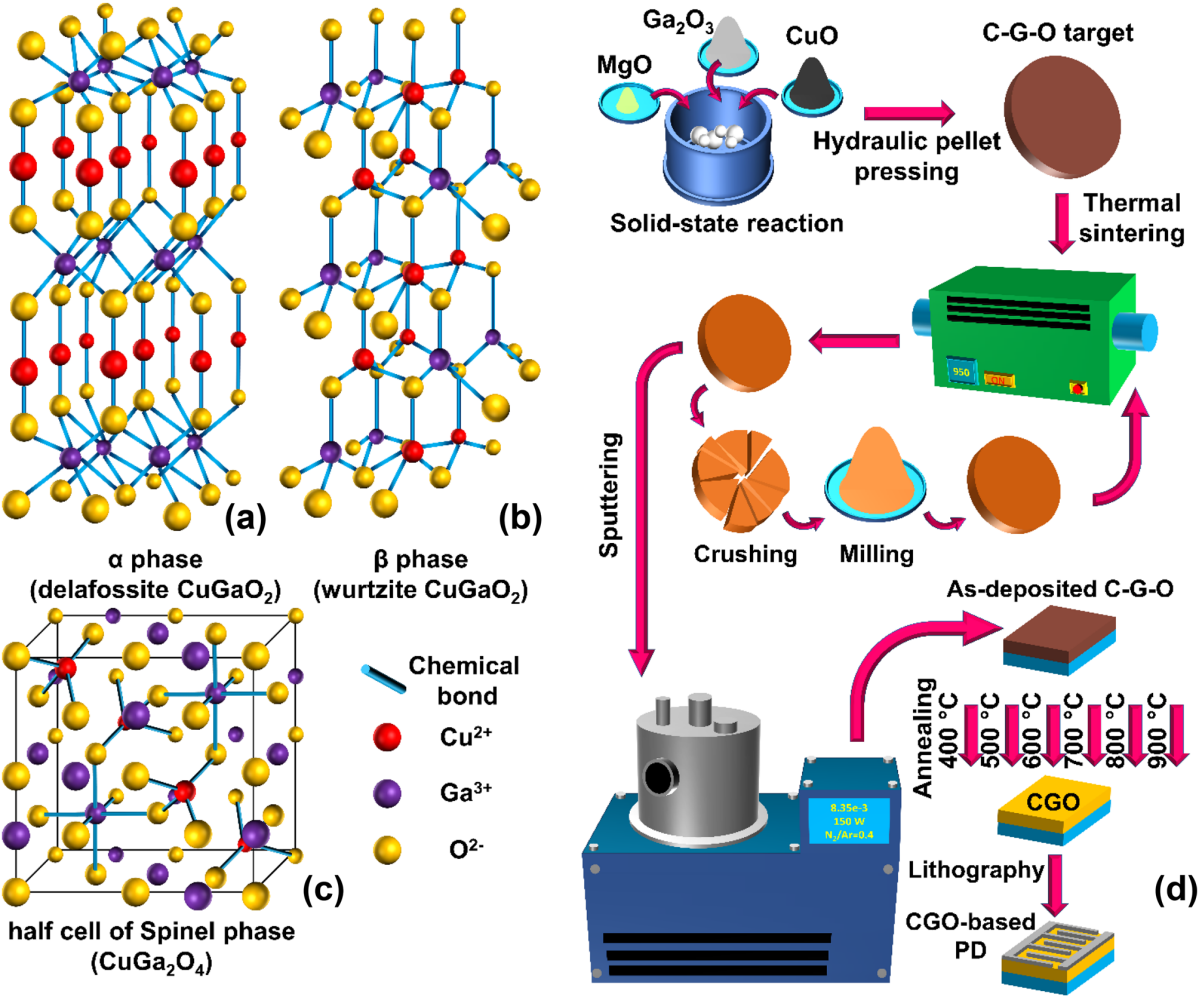
Schematic crystal structure of (**a**) α-CuGaO_2_, (**b**) β-CuGaO_2_, and (**c**) half-cell of spinel-CuGa_2_O_4_. The bigger spheres mean closer atoms. (**d**) Schematic route for experimental processes.

In general, β and α are two noteworthy phases of CGO material. The phase β, which has a wurtzite structure, is composed of vertex-sharing GaO_4_ and CuO_4_ tetrahedra and demonstrates a 1.47 eV bandgap^1^. Suzuki et al., point out that β-CGO is an appropriate option for manufacturing solar cells due to the high absorption coefficient and suitable direct bandgap^15^. CGO in α phase has a delafossite structure with $R\overline{3}m$ symmetry, in which the Cu atoms form a linear arrangement with O as O–Cu–O, while the Ga atoms create edge-sharing octahedra with O atoms. This atomic arrangement gives a periodic structure of Cu and GaO_6_ planes that reappear as ABCBAC stacking construction. Figure 1 compares the structures of delafossite (α-) and wurtzite (β-) CGO schematically. The α-CGO has a 3.6 eV bandgap, which uniquely distinguishes its properties from the β-CGO. Although, according to the classification, α-CGO is an indirect semiconductor, it has a direct transition with around 3.6–3.7 eV energy difference at points L and F (in k space), which may particularize the optical and electronic properties of this material. Suzuki et al., also mention that due to the wide bandgap and effective conductivity of α-CGO, it can be used as a suitable TCO^16^. Due to the lower formation energy of the delafossite phase compared to the wurtzite one, α-CGO is more stable while β-CGO is an unstable phase which can be decomposed to form α-CGO at temperatures higher than 460 °C^16^.

In addition to α and β phases, another phase which is formed as CuGa_2_O_4_ can also be realized for this material. This structure, which is called spinel phase, may be formed when the oxygen amount increases in the synthesis condition. Actually, when Cu and Ga contents are not well-controlled in the synthesis stage, additional phases such as spinel may be created^17^. The structure of this phase can be seen in Fig. 1c. As we can see, α-CGO includes GaO_6_ octahedra and β-CGO includes vertex-sharing CuO_4_ and GaO_4_ tetrahedra. The spinel phase is a combination of GaO_6_ octahedra and CuO_4_ tetrahedra that are together with a particular atomic arrangement.

In the last two decades, many attempts have been made to synthesize CGO in all the three phases of α, β and spinel. In recent years, β-CGO has been synthesized only with the help of the ion-exchange technique, meanwhile α-CGO has been attained by various methods such as hydrothermal, sol–gel process, sputtering, and solid-state reaction. The spinel-CGO is also obtained by methods such as electrospinning^18^, aerosol-assisted chemical vapor deposition (AACVD)^19^, chemical^20^, electrodeposition^21^ or decomposition from other phases. Considering the high thermal and chemical stability, as well as the appropriate optical and electronic properties of α-CGO, many electronic and optoelectronic devices can be built upon delafossite CGO.

α-CGO can be used in UV photodetectors. UV photoconductors have been thoroughly studied in recent years in order to use them in a variety of applications, including space and optical communication, astronomical studies, medical applications, ozone monitoring, combustion and flame detection, and so on. Numerous metal oxide semiconductors, including Ga_2_O_3_, ZnO, SnO_2_, and TiO_2_, have been studied for UV detection recently due to their affordability, robustness, light weight, and high responsivity^3,22–24^. Nowadays emerging materials based on copper oxide with delafossite structure of CGO can also be used in this field. The wide bandgap of the α-CGO semiconductor (3.6 eV), which is appropriate for UVA (320–400 nm) detection, optoelectronic and photovoltaic applications, is one of the most crucial essential parameters. α-CGO has a very high absorption coefficient (about 10^5^ cm^−1^), which helps to absorb more light in the UV range, while β-CGO lacks this feature. In addition, α-CGO shows high conductivity, which is a substantial advantage for a material with bandgap of 3.6 eV. Increasing conductivity considerably benefits to improve UV-PD responsivity. Lastly, the production of economical α-CGO is done easily by inexpensive and affordable methods with vast variety.

Today, Si-based technologies (CCD and CMOS) frame the majority of the UV-PD market. However, these technologies require technical complexities such as UV filters, which increase the weight and decline the performance of the system. Moreover, Si technology encounters restrictions at temperatures above 125 °C due to the thermal carrier generation, optical properties alteration, and device degradation under UV radiation^25^. Switching to wide bandgap semiconductors such as ZnO, Ga_2_O_3_, and CGO eliminates the essential filters for UV-PDs, as these materials inherently operate in UV range. Furthermore, the thermal carrier generation is insignificant in them due to the large bandgap. The UV-PDs based on these wide bandgap metal oxides are lightweight and require no cooling systems for minimizing the dark current, also demonstrate decent stability under ultraviolet radiation. ZnO is of great interest due to its high conductivity, affordability, non-toxicity and relatively low deposition temperature. Responsivity in ZnO-based UV-PDs is relatively high due to the substantial role of surface defects of oxygen vacancy. However, due to these defects and the mechanism of absorption/desorption of oxygen molecules on the surface, ZnO suffers from persistent photoconductivity (PPC)^26^. This denotes even after the optical signal is cut off, the current signal remains in the system for an extended time. Nevertheless, this phenomenon has not been observed in CGO. β-Ga_2_O_3_-based UV-PDs possess high gain due to self-trapped holes (STH) and subsequent photo-induced barrier lowering^22,27^. Yet, the transient response is slow in almost all the UV-PDs structures based on bulk crystal and homo or hetero-epitaxially grown β-Ga_2_O_3_ epi-layers^28^. This indicates the gain-bandwidth trade-off in these photodetectors is serious.

There are major challenges in the progress of CGO-based UV-PDs. The synthesis of α-CGO is still in the research phase and more investigation is required to fill this research gap. At the first step, the material quality of CGO, doping control and its uniformity in the synthesis stage should be improved in order to attain greater outcomes. This issue is more complicated in the UV-PD case. Extensive reports on various figure of merits (FOM) such as specific detectivity are not provided in most researches, and also the reliability of PDs is not investigated fairly. In addition, different research teams, including material scientists, device experts, and system engineers must work together to manage all aspects of the work.

In previous research^29^, we explored the electronic properties of α-CGO by investigating the Fermi level pinning (FLP) effect in this material and simulated an α-CGO-based UV-PD. Herein, we experimentally synthesize α-CGO films by a facile and scalable method and then investigate its structural, optical, and electronic properties. Thereafter, we fabricate a UV-PD by employing the synthesized material and carry out optical characterization of the device by preparing suitable metal electrodes in the form of Schottky as well as Ohmic contacts. In this way, we also inspect the influence of the metal contact on the performance of the PDs made from α-CGO.

## Experimental methods

Solid-state reaction method was used to synthesize the CGO target. In this route, stoichiometric proportions of Ga_2_O_3_ and CuO were combined and reacted in a ball milling system for 48 h. According to the experience of our previous research^30^, which showed that adding 2.5% Mg impurity to the CuCrO_2_ (CCO) greatly increases the conductivity, an appropriate amount of MgO was also added to the reaction cups. The radius of divalent Mg^2+^ ions is to some extent bigger than that of Ga^3+^ ions. By the way, the atomic radii of Mg^2+^ dopants and their host atoms (Ga^3+^) are not too dissimilar to cause strong disorder in the crystal structure at low doping levels^31^. However, the presence of Mg impurity can create a slight strain in the CGO structure. Renaud et al., showed that more than 5% Mg doping causes disordered structure and also reduces the semiconductor conductivity^32^. Therefore, we preferred only 2.5% Mg doping in all the synthesized samples.

After the production of C–G–O powder (an amalgam of Cu, Ga, and O atoms without delafossite phase), it was turned into a 2" (~ 5 cm) tablet under 30 MPa pressure and was sintered in a furnace under N_2_ atmosphere at 950 °C temperature for 12 h. The prepared C–G–O target once again was crushed, milled, and heated at 950 °C in N_2_ atmosphere. This process was repeated for 2 more times. After forming an appropriate target, thin film deposition was proceeded by sputtering method. 1.5 cm × 1 cm quartz substrates were washed by deionized water, ethanol, and acetone in an ultrasonic washing machine for 20 min. The substrates were transferred to the sputtering chamber at initial 3 × 10^−5^ mbar vacuum. Using Ar and N_2_ gas with N_2_/Ar = 0.4 and 8.35 × 10^−3^ mbar pressure, the sputtering procedure was set up. The sputtering power was set to 150 W for 2 h deposition process. The sputtering parameters were summarized in Table 1. After the deposition step, the samples were subjected to heat treatment to attain the appropriate delafossite phase. The sputtered samples were annealed at different temperatures of 400, 500, 600, 700, 800 and 900 °C under 10^−5^ mbar vacuum for 2 h.

**Table 1:** The parameters values in sputtering process.

Items	Conditions
Base pressure	3.0 × 10^−5^ mbar
Deposition pressure	8.35 × 10^−3^ mbar
Sputtering gas ratio (N_2_/Ar)	0.4
Power	150 W
Target-to-substrate distance	60 mm
Deposition time	120 min
Substrate temperature	300 °C
Substrate	Quartz

In order to fabricate the CGO-based UV-PDs, silver (Ag), copper (Cu), and nickel (Ni) targets were ablated using Nd:YAG laser (532 nm wavelength, 10 ns pulse duration) with 10 Hz pulse repetition rate and 250 mJ energy per pulse in vacuum chamber to form a 100 nm metallic coverage on top of the CGO thin films. The target-to-substrate distance was adjusted with respect to the size of plume and the CGO layer was kept away from the brightest part of the plume. Consecutively the interdigitated contacts were applied on the metal layers employing standard photolithography technique followed by wet chemical etching. Figure 1d schematically illustrates the experimental processes to fabricate CGO-based UV-PD from the first step to the last.

With the aim of inspection, the crystal structure of the prepared CGO layers, XRD analysis was arranged for all the samples by X-ray diffractometer (XRD, STOE STADI-P Co., Germany). The absorption spectra of the thin films were investigated by an Avantes spectrophotometer (Avaspec-3648, Netherland) and the optical bandgap of the synthesized samples was obtained by the Tauc plotting method. In order to further investigate the crystal and molecular structure of the synthesized samples, Raman and FTIR spectra were prepared by Teksan (Takram P50C0R10, 532 nm), and BOMEM (MB-series) spectrometers. The surface morphology as well as the cross-section images of the prepared films were captured and analyzed by field emission scanning electron microscopy (FESEM, Mira III, TeScan, Czech Republic). Moreover, to find out the atomic composition, the installed EDS module on this device was operated. In addition, electrochemical impedance spectroscopy (EIS) and Mott-Schottky analysis were used by a potentiostat device (IVIUM, The Netherlands) in the frequency range of 1 Hz–1 MHz to investigate the electrochemical and electronic properties of the samples. In these analyses, 0.1 M Na_2_SO_4_ electrolyte solution, platinum wire counter electrode and Ag/AgCl reference electrode were applied. Lastly, for the characterization of the UV-PD devices, a 2450 Keithley source measure unit (SMU) was used to examine the current–voltage characteristic curves of the devices as well as the transient behavior of the PDs. We used a 365 nm LED (Donggum Hongke Lighting Co., LTD) in photodetection setup. the LED was placed at a distance of 10 mm from the UV-PDs and a probe station with two gold test probes are employed to connect the electrodes. The LED ON/OFF operation was controlled by a Chroma 62012P-600–8—Programmable DC Source device.

## Results and discussion

**Figure 2:**
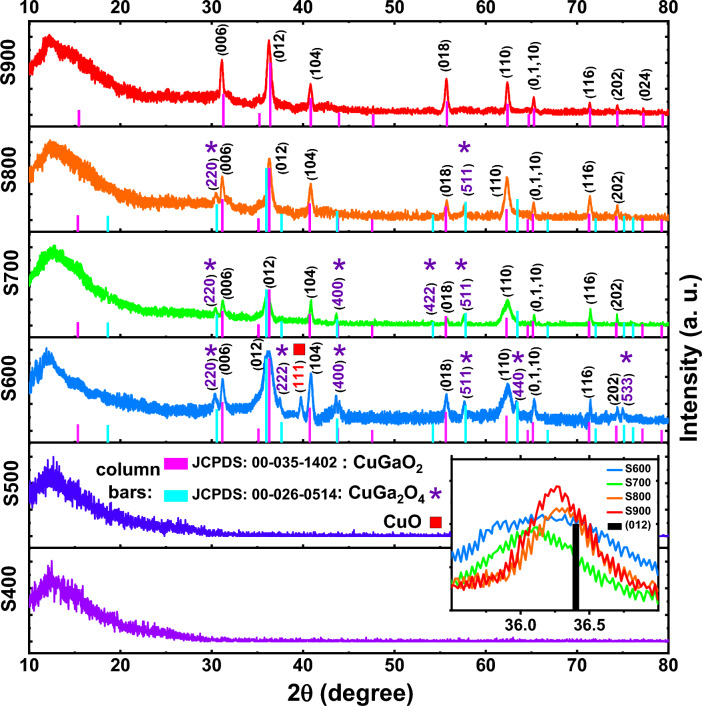
XRD patterns of the samples annealed at different temperatures. The column bars stand for main diffraction angles of delafossite CuGaO_2_ (α-CGO) and spinel CuGa_2_O_4_. In the inset the zoomed-in pattern demonstrates slight shifts for XRD patterns compared with the standard card.

Figure 2 illustrates the XRD patterns for samples annealed at different temperatures, which are now named S400, S500, S600, S700, S800, and S900. S400 and S500 samples have no characteristic peaks and only include a background peak at 12° due to the quartz substrate. In this sense, the mentioned samples do not have a specific crystal structure and are only amorphous films. In other words, the Cu, Ga, and O atoms from the C–G–O target sits on the quartz substrate in a disordered configuration after sputtering. But in low temperatures, the necessary reactive energy is not high enough to form the delafossite phase^33^. It has been revealed that pure copper-based delafossite is attained at high temperatures^34^. As Yu et al.^6^ pointed out, the delafossite CGO is formed only at temperatures higher than 600 °C. Yu and Lee^33^ also found that the pure delafossite CGO is formed at temperatures higher than 750 °C.

As can be seen from Fig. 2, the XRD pattern for S600 sample illustrates the peaks at 31.17, 36.16, 40.84, 55.71, 62.37, 65.30, 71.47, and 74.39°, which can be corresponded to (006), (012), (104), (018), (110), (0 1 10), (116) and (202) crystal planes of delafossite structure, respectively. These peaks can be attributed to the rhombohedral structure with $R\overline{3}m$ space group according to JCPDS standard card, 00–035-1402. In this sample, the presence of some additional peaks can also be seen, which are located at the diffraction angles of 30.37, 37.45, 39.75, 43.66, 57.65, 63.35, and 74.98°, respectively. According to PDF2 standard cards, 00–026-0514 and 00–001-1117, these peaks can be associated with spinel phase (CuGa_2_O_4_) and CuO. Shi et al.^17^ and Ahmed et al.^35^ separately indicate that the spinel phase is usually gained at high oxygen condition. Shi et al.^36^, also claim that sputtering and sol–gel methods do not always yield pure CuGaO_2_ phase and secondary phases with different orientations may be observed in the samples. Despite the absence of oxygen in the chamber before deposition, spinel phase has been formed and we believe that the CuO precursor in the sputtering target causes this. Most synthesis routes, comprising solid-state reaction for delafossite CGO, exploit Cu_2_O^16,37,38^, which includes reduced amount of oxygen in its structure than CuO. The presence of excess oxygen causes the partial formation of spinel phase in S600 and S700 samples. In the annealed samples at higher temperatures, the spinel phase becomes less and less and completely disappears in S900 sample. Saikumar et al.^34^, also showed that the spinel phase is formed through the sputtering method at a temperature lower than 800 °C. Varadarajan et al.^39^ showed that the spinel phase is actually a meta-stable phase that forms at low temperatures and practically disappears at temperatures above 700 °C. In this sense, the formation of pure delafossite phase without impurity and additional spinel phase at 900 °C can be realized. Also, Yu et al.^6^ pointed out that delafossite CGO is unstable at temperatures below 600 °C, and the oxidation of Cu^I^ at lower temperatures causes decomposition of delafossite into CuO and spinel CuGa_2_O_4_.

It is clear that the (012) peak has become sharper at higher temperatures. According to Debye–Scherrer equation, $D=k\lambda /\beta cos(\theta )$ ^40^, where $k$ is a constant, $\lambda$ is the wavelength of X-rays, $\beta$ is the full width at half maximum (FWHM) of the peak, and $\theta$ is the diffraction angle, it can be concluded that by reducing the FWHM of the (012) peak, the crystallite size ($D$) of S900 turns out to be larger than that of the S600. This feature and the absence of additional phases help to increase the conductivity in S900 sample. The presence of additional spinel GuGa_2_O_4_ in S600 and S700 causes the formation of grain boundaries and as demonstrated by Tsay and Chen^41,42^, these boundaries by some means reduce the mobility and carrier concentration and eventually decrease the conductivity in the material.

The existence of Mg is not obviously recognized, which means that it does not form a new phase and no additional peak is seen in the samples associated to MgO or Mg compounds. Li et al.^31^, demonstrated that the main peaks shift to lower angles by Mg doping in CGO structures. To clarify this issue, in addition to the main lines of the JCPDS: 00–035-1402 (α-CGO) and 00–026-0514 (spinel-CGO), we have illustrated a zoomed-in XRD pattern of the samples in the range of 35.5–37.0° (Fig. 2 inset) which shows a slight shift towards lower angles. The presence of divalent Mg^2+^, which usually replaces trivalent Ga^3+^, causes strain in the CGO structure, which in turn, shifts the XRD peaks to the lower angles^43^. So, it can be concluded that Mg is effectively doped into the CGO structure. The atomic radius of Mg^2+^ is slightly larger than that of Ga^3+^. Therefore, by replacing Ga^3+^ with Mg^2+^, the larger Mg^2+^ ions push slightly the crystal structure, which causes compressive strain. As we know, compressive strain moves the XRD pattern peaks to lower angles^44^. At higher annealing temperatures, atoms move due to the diffusion phenomenon and the density of doping atoms changes in local areas. However, our purpose is not to examine these phenomena in detail. We have tried to choose a proper dopant to increase the carrier concentration in the CGO material and provide the desired conductivity.

**Figure 3:**
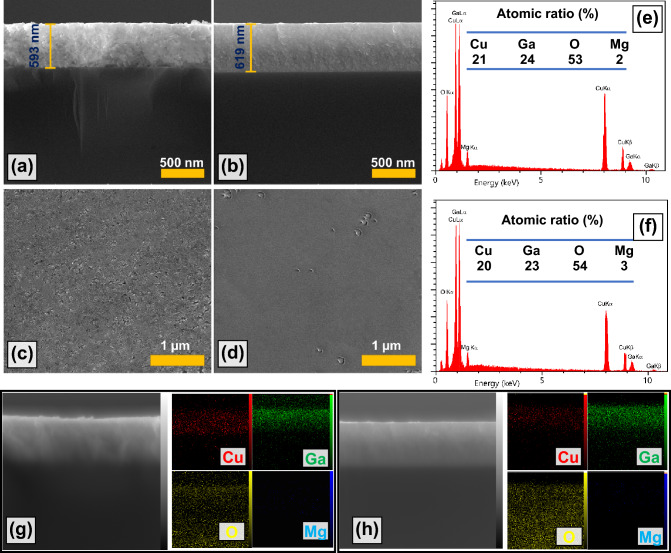
Cross-sectional FESEM images for (**a**) S600, and (**b**) S900 thin films. Top view FESEM images captured from (**c**) S600, and (**d**) S900 samples. EDS with associated atomic ratio tables for (**e**) S600, and (**f**) S900 samples. EDS mapping acquired from cross-section of (**g**) S600, and (**h**) S900 layers.

We prepared cross-sectional and top view FESEM images of the S600 and S900 samples to investigate the quality and morphology of the synthesized thin films, which can be seen in Fig. 3. Figure 3a, b show the cross section of S600 and S900, respectively. The thickness of the samples is almost the same of around 600 nm, and the uniformity of the samples can be well observed in both samples. Figure 3c, d represent the top view of S600 and S900 samples, respectively. In both samples, the uniformity of the surface can be clearly seen, which indicates the high quality of the samples. A major difference can be seen in the FESEM images of the two samples. The S900 sample has a more uniform morphology, and it seems that with formation of the single-phase CGO, grains have sunk into each other and formed a uniform structure. On the contrary, in S600 sample, with the presence of additional spinel phases, the morphology is grainy.

In addition to the FFESEM images, the distribution of atomic elements in the S600 and S900 samples can be seen as EDS analyses in Fig. 3e, f. The cross-sectional EDS mapping of these samples are also available in Fig. 3g, h. The results of these analyses are summarized quantitatively in the tables of Fig. 3e, f. Cu, Ga, O and Mg elements can be observed in both samples. The atomic ratio of Mg element in the two samples is around 2–3%, which is almost consistent with our applied conditions in the synthesis stage. It is possible that EDS results are not very reliable for low thicknesses. Thus, in the course of our inspections, we examined several layers with various thicknesses. We synthesized the CGO layer prepared at a temperature of 900 °C even with a thickness of more than 1 μm. The cross-sectional FESEM image along with the EDS spectrum of this layer can be seen in Figure S1 of the supplementary information. The EDS spectrum of this relatively thick CGO layer (more than 1 µm) indicates the stoichiometric ratios of the various elements are similar to those we have reported for the 600 nm layers. For the thickness above 1 μm, the atomic ratio of Mg is estimated to be between 2 and 3%, which is almost in the same range in the 600 nm samples. There is a similar analogy for oxygen atomic ratio. The existence of this stoichiometric similarity led us to report atomic ratios for the 600 nm layers as well. Based on the specific doping of our target (2.5%), we expect the atomic percentage of Mg in all the prepared layers (600 nm and 1100 nm) to be almost equal. In case of changes in oxygen atomic ratios in thinner layers on a substrate, it is possible the amount of Mg also encounters wide changes. Such an alteration in the atomic ratio of Mg has not been observed.

The relative concentration of Cu compared to the stoichiometric state (Cu_1_Ga_1_O_2_) shows around 4 and 5% Cu vacancy (V_Cu_) in S600 and S900, respectively. In fact, V_Cu_ is one of the most important p-type conductivity factors in the delafossite CGO^45^. Gake et al.^46^ showed that the formation energy of V_Cu_ is lower than that of all the other defects (e.g. Cu_Ga_, Ga_Cu_, O_i_, V_O_, Cu_i_, Ga_i_, and V_Ga_). This easily-formed defect causes shallow acceptor levels and p-type conductivity in CGO. The intrinsic acceptor-like defects, V_Cu_, have a very low or even negative formation energy in some cases (at high Fermi energies), which cause the compensation of electron carriers and completely rule out the n-type CGO. Bredar et al.^5^ exhibited that the hole concentration in CGO can increase up to 10^21^ cm^−3^ by only 4% Cu vacancy. In addition to V_Cu_ (and oxygen interstates, O_i_), intentional doping with divalent atoms in CGO structure can increase the hole concentration. Li et al.^31^, Herraiz-Cardona et al.^47^, and Tsay et al.^42^ investigated Mg doping in CGO structure and showed that the hole concentration and conductivity can be controlled by Mg doping. Moreover, excess V_Cu_ in CGO:Mg has been observed by Bredar^5^. This means that Mg doping increases the V_Cu_ itself, and elevates the hole concentration.

**Figure 4:**
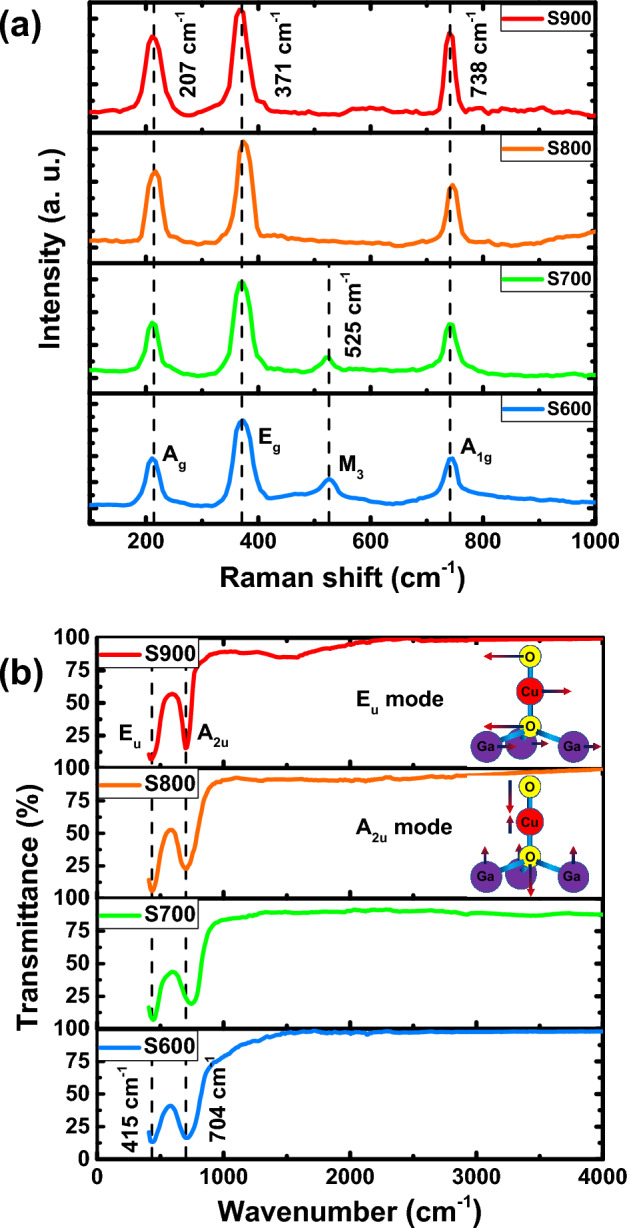
(**a**) Raman, and (**b**) FTIR spectra for the samples annealed at different temperatures. In the inset E_u_ and A_2u_ vibrational modes of delafossite structure are depicted.

To study the structural features and chemical bonds formed in the thin films, we prepared Raman and FTIR spectra. Raman spectra of samples S600, S700, S800 and S900 are depicted in Fig. 4a. Irreducible representation of phononic modes for delafossite CGO at the $\Gamma$ point of Brillouin zone is as^48^:

1 $$\Gamma = A_{1g} + E_{g} + 3A_{2u} + 3E_{u}$$

In this representation, $A$ modes represent lattice oscillations along the c-axis (along the O–Cu–O bonds), while the double degenerate $E$ modes represent oscillations perpendicular to the c axis. The g-indexed modes $\left({A}_{1g}+{E}_{g}\right)$ are even modes and Raman active, while u-indexed $\left({A}_{2u}+{E}_{u}\right)$ odd modes (both oxygens oscillate in phase) are not Raman active and only IR active. In the ${A}_{1g}$ mode, Cu and Ga atoms are fixed, and O atoms oscillate in the opposite phase to each other along the c-axis. The observed peaks at 371 and 738 cm^−1^ in Fig. 4a can be attributed to ${E}_{g}$ and ${A}_{1g}$ vibration modes. Ahmed and Mao^49^ reported similar observations in their research. The peak observed at 207 cm^−1^ also indicates the ${A}_{g}$ vibration mode. Jlaiel et al.^50^, by investigation of delafossite CGO pointed to this peak, which is caused by the *σ* plane symmetry. As previously mentioned, by increasing temperature and growing crystallite size in S900 sample, it has become more uniform. Therefore, the Raman width has decreased in ${E}_{g}$ and ${A}_{1g}$ peaks, which is in good agreement with the XRD results. In the S600 and S700 samples, minor peaks are also seen in 525 cm^-1^, which can be attributed to M_3_ mode. This mode, which is a stress-induced effect of ${A}_{g}$ and/or ${B}_{u}$ modes, can be caused by the strain effects of spinel and CuO phases. According to the Raman selection rule, these modes are forbidden, although, they will be allowed by symmetry breaking^51^.

Figure 4b demonstrates the FTIR spectra for the samples. ${E}_{2u}$ and ${A}_{2u}$ vibration modes which are inactive in Raman analysis are active in FTIR now. For better understanding, the oscillation models of these modes are depicted in the inset of Fig. 4b. The peaks observed in all the samples around 415 and 704 cm^−1^ represent ${E}_{u}$ and ${A}_{2u}$ modes, respectively. These peaks stand for O–Cu and O–Ga bonds, which have been confirmed previously^4,52^. Although, the presence of Mg cations does not create a new peak, it causes the FTIR peaks to shift to higher wavenumbers due to lower mass of Mg compared to Ga^43^. Using ab-initio calculations, Pellicer-Porres et al.^48^ calculated the oscillation frequencies of ${E}_{u}$ and ${A}_{2u}$ modes as 387 and 645 cm^−1^, respectively, which are clearly lower than our observations. The presence of spinel phase in FTIR analysis and distinguishing it from delafossite phase is impossible because the frequency range of its FTIR modes completely overlaps with those of the delafossite^53^. Raman analysis, along with FTIR, shows the quality of the crystal structure and the type of molecular bonds. They confirm that the CGO crystal structure is well-formed in S900 sample, because the peaks have become sharper as the temperature rises. This means that a finer crystal structure has been configured, which is in complete agreement with the XRD and FESEM results.

**Figure 5:**
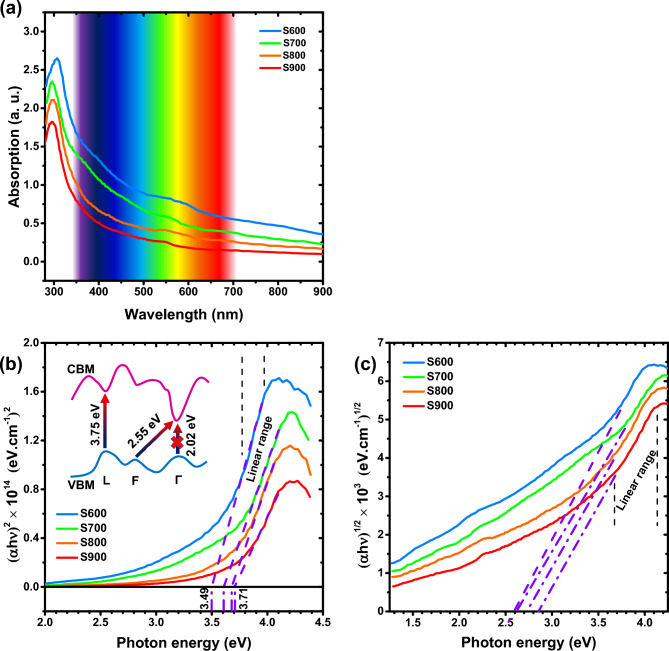
(**a**) absorption spectra for the CGO thin films synthesized at different condition. Tauc plots of according samples by (**b**) direct $(\mathrm{n}=1/2)$, and (**c**) indirect $(\mathrm{n}=2)$ bandgap consideration. All the fittings are exerted in linear ranges of the plots by OriginLab software. At the inset a schematic curve illustrates the conceptualized band structure for α-CGO and some main electron transitions with different energy distance.

With the purpose of studying the optical properties of the synthesized thin films and evaluating their optical bandgap, we provided absorption spectrum from the samples, which can be seen in Fig. 5. Figure 5a specifies the absorption spectra for different samples. As can be realized from the figure, the absorption edge of the samples is found to be undergone a blue shift with the temperature increment, which indicates the alteration in optical bandgap. Using the Tauc Equation^54^;

2 $$\alpha h\nu = A\;\left( {h\nu - E_{g} } \right)^{n}$$

the optical bandgap can be calculated. In this equation, $\alpha$ is the absorption coefficient, $h\nu$ is the photon energy, $A$ is an energy independent constant, ${E}_{g}$ is the bandgap, and $n$ is equal to 1/2 for direct bandgap and 2 for indirect one. Figure 5b displays the Tauc plots with $n$ =1/2 (direct bandgap) for different samples. By fitting the curves in the linear range, the direct bandgap for S600, S700, S800 and S900 samples can be evaluated as 3.49, 3.61, 3.68, and 3.71 eV, respectively. The bandgap increases with temperature, which was also reported by Yu et al.^33^. In general, the tensile strain reduces the bandgap^55^. It can be predicted that, as the temperature declines, the delafossite/spinel boundaries cause tensile strain and the bandgap decreases. Additionally, the spinel-CGO, which is found more in S600 and S700 samples, has an indirect bandgap of 1.77 eV^56^. In this sense, the slight decrease in bandgap in samples with lower annealing temperature can be related to this factor to some extent.

The absorption spectrum visible region tails, which occurs for all the samples (Fig. 5a) can be attributed to the substitution of Ga^3+^ by Mg^2+^ cations. In this case, some defect levels are infiltrated into the energy gap, which cause alteration in the absorption curves^2^. Li et al.^31^ have also observed this phenomenon by Mg and Zn doping. Based on their explanation, the presence of Mg and Zn impurities causes the injection of hole carriers into the CGO, which leads to more transitions in the band structure as well as strong absorption in the visible region.

According to density functional theory (DFT) calculations using the local density approximation including the Hubbard correction (LDA + U), the α-CGO is practically an indirect semiconductor^16^. There are L-L, Γ-Γ and F-Γ transitions with corresponding energies of 3.75, 2.02 and 2.55 eV. The L-L and Γ-Γ transitions are direct and the F-Γ transition is indirect, even though Γ-Γ transition is forbidden. The indirect phonon-assist F-Γ transition with 2.55 eV energy difference, have inferior absorption cross-section, so occurs with minor probability. However, these indirect transitions can also be effective in the tail-shape of the absorption spectra. As it known, the absorption curve of an indirect semiconductor is not very sharp and always grows with a mild slope. Figure 5c shows the Tauc plot considering $n$ =2 (indirect bandgap) for the samples. The indirect bandgaps of CGO can be obtained by fitting the curves in the linear region. These bandgaps are lied between 2.6–2.8 eV, which is in good agreement with our earlier discussion.

We measured the DC conductivity of the layers synthesized in different conditions, using a 4-point probe device. In this setup, 4 golden tip terminals were placed at equal distances of 1 mm on the CGO layers. To increase the accuracy of the system, the two inner and outer probes separately read the voltage and current, respectively. Table 2 summarizes the DC conductivity for the samples. According to the dynamic range of the 4-point probe system, the two samples S400 and S500 showed over-loaded resistance that indicates a very low conductivity of these samples, which could not be measured. The conductivity of S600, S700, S800 and S900 samples is 1.19 × 10^−3^, 1.81 × 10^−3^, 1.86 × 10^−3^ and 2.24 × 10^−3^ S/cm, respectively. The conductivity of the layers increases with annealing temperature. These values are close to the results of the other groups that have investigated the α-CGO conductivity, which are also included in Table 2. Conductivity test results are in good agreement with previous outcomes. As discussed earlier, the samples synthesized at lower temperature were multi-phase, and by increasing the annealing temperature to 900 °C, the pure delafossite phase was attained. The secondary phases and formation of electron scattering centers at the boundaries of different phases can lead to decrease in the conductivity, which is in good agreement with the results of the DC conductivity test.

**Table 2:** DC conductivities measured by 4-point probe device for S400–S900 samples (the table also contains some other reports from literature).

Sample	DC conductivity σ (S/cm)	Ref.
S400	–	This work
S500	–	This work
S600	1.19 × 10^−3^	This work
S700	1.81 × 10^−3^	This work
S800	1.86 × 10^−3^	This work
S900	2.24 × 10^−3^	This work
Plasma assisted reactive evaporated p-CuGaO_2_	1.45 × 10^−3^	^63^
Sputtered delafossite p-CuGaO_2_ thin film	1.22 × 10^−7^–4.30 × 10^−3^	^86^
Sputtered delafossite p-CuGaO_2_ thin film	8.22 × 10^−3^–1.74 × 10^−2^	^33^
Sol–gel derived CuGaO_2_ thin film	2.82 × 10^−5^–7.41 × 10^−3^	^41^
Sintered pellet CuGaO_2_ (after solid state reaction)	1.50 × 10^−6^–3.30 × 10^−3^	^38^
Spin coated p-CuGaO_2_	3.05 × 10^−3^–1.07 × 10^−2^	^42^

**Figure 6:**
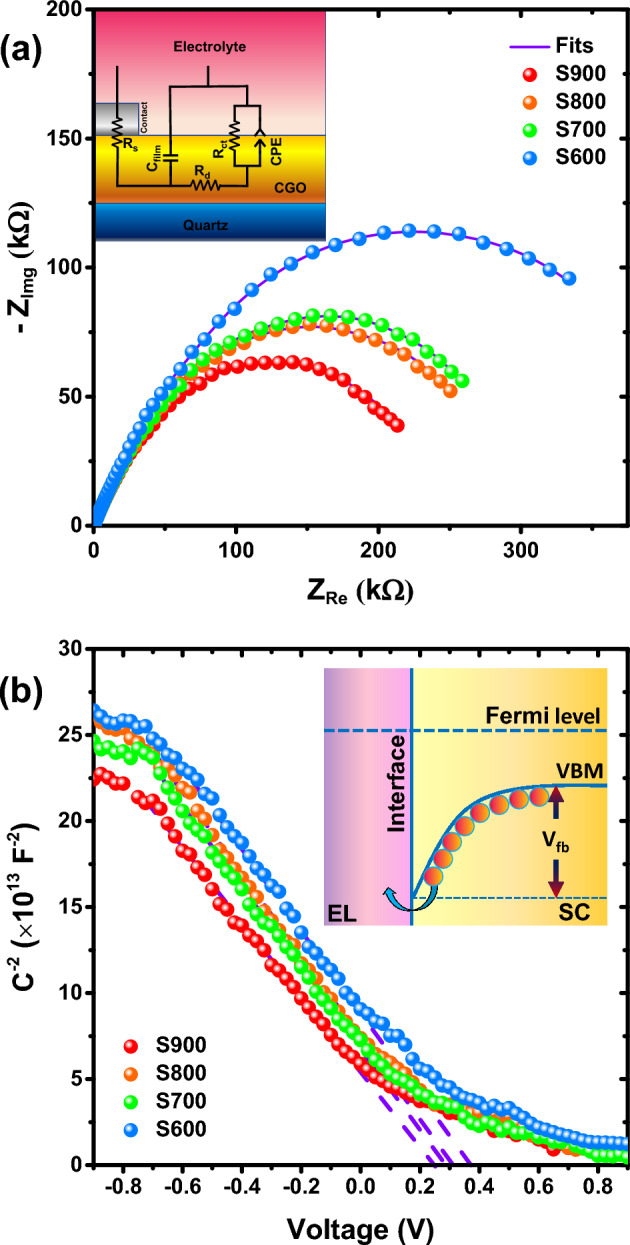
(**a**) Nyquist plots, and (**b**) Mott-Schottky analysis for S600-S900 samples attained by a conventional three-electrode electrochemical cell in Na_2_SO_4_ electrolyte. In the inset the applied equivalent circuit and the concept of flat-band voltage are depicted.

To further investigate the electron transport properties of the synthesized samples, we arranged the EIS analysis. This setup was included a CGO thin film as the working electrode, Ag/AgCl reference electrode and a platinum counter electrode. The frequency was modulated in the range of 1 Hz to 1 MHz. The analysis was performed in the dark mode with a 0.9 V large-signal bias and a small-signal voltage of 10 mV. The Nyquist plots of different samples are shown in Fig. 6a. The employed modified Randles equivalent circuit for fitting the experimental results can be seen in the inset. The Nyquist plots of all the samples consist of a semicircle, which indicates the presence of only one interface junction (CGO/electrolyte). In the equivalent circuit of such Nyquist plot, R_s_ is the series resistance and represents all the resistances in the external circuit, including the electrolyte and contacts resistance. R_d_ and C_film_ characterize the charge transport resistance and the thin film capacitance, respectively. R_ct_ stands for the charge transfer resistance at the CGO/electrolyte interface and CPE is a constant phase element representing the capacitance caused by the depletion region at this interface. The charge transport resistance (R_d_) in S600, S700, S800, and S900 samples is 476, 331, 307 and 260 kΩ, respectively. As can be seen, by the annealing temperature increment, the charge transport resistance decreases, which agrees with our previous observations.

Figure 6b shows the curves of Mott-Schottky analysis for S600, S700, S800, and S900 working electrodes achieved by the same setup as the EIS analysis. The results were measured at frequency of 1 kHz, in the voltage range of − 0.9 to + 0.9 V. Considering the negative slope of the plots, it can be recognized that all the samples are p-type semiconductors. According to the slope of the curves, x-intercepts, and Mott-Schottky relationship:

3 $$\frac{1}{{C^{2} }} = \frac{2}{{\varepsilon \varepsilon_{0} A^{2} eN_{d} }} \left( {V - V_{fb} - \frac{{k_{b} T}}{e}} \right)$$

One can find the hole concentration and the flat-band voltage. In this equation, $C$, $\varepsilon$, and ${\varepsilon }_{0}$ stand for measured capacitance by the potentiostat, relative permittivity, and vacuum permittivity, respectively. $A$ and $e$ denote the film active area in contact with electrolyte and elementary charge, correspondingly. ${N}_{d} ,V$, and ${V}_{fb}$ represent acceptor concentration, applied potential by potentiostat and flat-band voltage, respectively. ${k}_{b}$ and $T$ are Boltzmann constant and the absolute temperature (~ 300 K). Based on the complete ionization approximation at room temperature, it can be concluded that the hole concentration is almost equal to the acceptor concentration. In this case, the hole carrier density for S600, S700, S800, and S900 samples is 7.87 × 10^16^, 8.55 × 10^16^, 7.81 × 10^16^, and 8.98 × 10^16^ cm^−3^, respectively. Although, the hole concentrations are close to each other, the hole concentration in S900 sample is higher than that of the other samples, and there is no clear trend in carrier density of the samples. The relative higher hole concentration in S900 sample can help to increase the conductivity in this sample, and as we have seen before in the EIS and DC conductivity analyses, this sample showed a higher conductivity than that of the other samples. The ${V}_{fb}$ is estimated using the x-intercepts for S600, S700, S800, and S900 samples, respectively as 0.37, 0.28, 0.31, and 0.24 V. The flat-band voltage is the potential barrier against the flow of charge carriers from the semiconductor to the interface. The inset of Fig. 6b illustrates the concept of the flat-band voltage. Even though, ${V}_{fb}$ is obtained for the CGO/electrolyte interface, it is possible to compare the flat-band voltages between different samples clearly. Among all samples, S900 has the lowest ${V}_{fb}$. In this sense, at a metal/S900 junction, we can expect a lower flat-band voltage and the current flow can stream with reduced difficulty.

**Figure 7:**
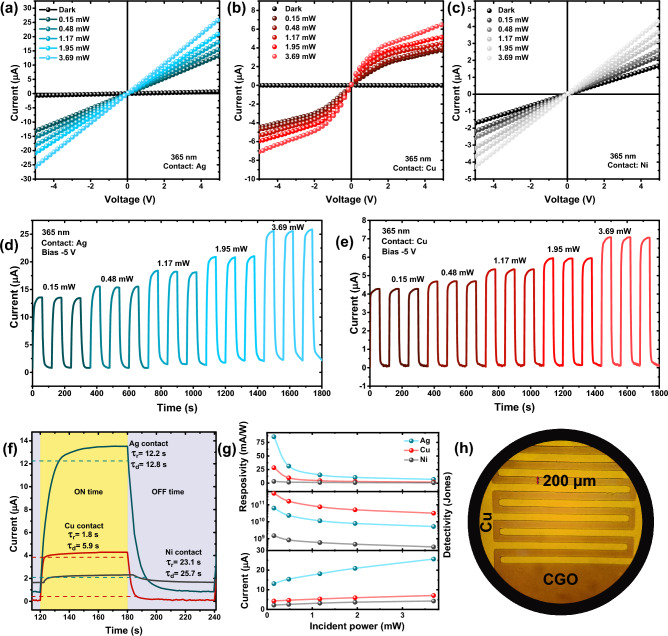
The I–V characteristics for (**a**) Ag-, (**b**) Cu-, and (**c**) Ni-based contact PD under different incident power (365 nm). The I–T curves of the (**d**) Ag, and (**e**) Cu PD devices. (**f**) PD transient behavior for Ag, Cu, and Ni contact samples between ON and OFF states. (**g**) Power-dependent parameters of responsivity, detectivity, and photocurrent for different fabricated devices. (**h**) Microscopic image of the interdigitated Cu fingers on the CGO layer.

After the heat treatment and formation of α-CGO thin films, we fabricated an MSM-type photoconductor detector with the same electrodes. For this purpose, we deposited copper (Cu), silver (Ag), and nickel (Ni) metals by PLD method on the samples and then patterned interdigitated electrodes with 200 μm distance by photolithography technique. Various metal contacts with different work functions can change the characteristics of the photodetector. For example, depending on the metal electrode work function, the contact can be Ohmic or Schottky, which changes the photovoltaic properties of the device. We chose three metals, Cu, Ag and Ni, which have approximately work functions of 4.5, 4.7 and 5.1 eV, in order to investigate their effect on the UV-PD by having a range of work functions. Figure 7h represents the microscopic image of these electrodes on the CGO thin film. We examined the performance of fabricated samples with different electrodes, using 365 nm light illumination and a Keithley source-meter. We employed only S900 sample to fabricate the PDs due to superior electronic characteristics and pure delafossite phase of this sample.

Figures 7a, b, c show the I–V characteristics at different incident powers for the S900 sample with three Ag, Cu, and Ni electrodes. The I–V curves for the Cu sample demonstrate the characteristic curve of a Schottky-type MSM PD. The photodetectors with Ag and Ni electrodes perform as Ohmic devices. Considering the low work function of Cu (4.5 eV), this result could have been expected. As a general rule, if the work function of metal is lower than that of the p-type semiconductor, the metal/semiconductor contact will be a Schottky one^57^. However, the exact knowing of work functions may not help to understand the connection nature correctly, because various and complex factors are involved in this phenomenon. For example, the Fermi level pinning effect can change the nature of connection to Schottky or Ohmic, contrary to the Schottky–Mott rule. Determination of Ohmic or Schottky contact according to the work function difference is a general statement. But as a whole, the low work function of copper compared to what we expect from CGO (4.9–5.3 eV) gives us an a priori expectation that the contact must be Schottky. In the case of nickel, which has larger work function, we expect Ohmic contact. However, as mentioned above, knowing the exact work functions (electrodes and semiconductor), it is not possible to make a definite decision about the type of connection with complete certainty. We believe the best judgment about the contact nature can be deduced from Figs. 7a, b, c, which are taken from the experimental results. Differences in work functions can be meaningful, but this is not the whole story.

As we mentioned in our previous research^29^ on simulated CGO-based PDs, the behavior of the system for metal work functions lower than 5.1 eV cannot be Ohmic. However, in the current research, the Ag electrode with the work function of 4.7 eV exhibits linear behavior. It should be noted that some definite phenomena were ignored in our simulation. In fact, some actual conditions cannot be investigated using TCAD simulation. For instance, in the process of establishing a metal contact on the CGO, a large number of Ag atoms penetrate into CGO, and despite the fact that the ideal case of Ag contact is Schottky, the contact becomes Ohmic by reducing the CGO work function, and/or by reducing the resistance of the interface. This trend has been reported in several articles^58–62^. Moreover, in our previous research, it was calculated that the CGO work function is between 5.30 eV and 5.64 eV. In that case the contact was Schottky-type, assuming a pure Au electrode with a work function of 5.10 eV. It should be mentioned that different synthesis conditions and methods produce CGO with wide work function range. For example, Esthan et al.^63^ have succeeded in synthesizing CGO by oxygen plasma assisted reactive evaporation technique with the work function of 4.9 eV, which is much closer to the Ag work function (4.74 eV). Therefore, the possibility for Ohmic contact is not far from mind.

The behavior of the photodetectors can be described as follows for different metal electrodes. In dark state, some holes are generated due to thermal excitations in the CGO semiconductor. By applying a bias voltage, these holes drift by the electric field and are collected in the electrodes, leading to the formation of dark current. By illuminating light, excess carriers are generated in the semiconductor bulk. Due to their Ohmic contact nature of photodetectors based on silver and nickel electrodes, the Schottky barrier is infinitesimal against the flow of electrons and holes, and they can be easily collected by the electrodes. A higher bias voltage applies a higher electric field to the CGO, which causes a higher current to flow in the photodetector, corresponding to the voltage bias. In this way, the behavior of the I–V curve in silver and nickel samples will be linear or, in other words, Ohmic. However, the behavior of the photodetector based on the copper electrode exhibits a different behavior. Copper, having a lower work function, causes a nearly large Schottky barrier. The Cu/CGO/Cu structure forms a two back-to-back Schottky diode system. By applying voltage bias to this system, one diode is always in forward bias and the other one is in reverse bias. The width of depletion layer for the electrode in forward bias is reduced and the carrier flow is easily possible. However, in the reverse biased electrode, the width of depletion layer becomes large. In this way, almost all the applied voltage exerts on the depletion layer of the reverse biased electrode. Actually, the total current of the photodetector is limited by the reverse biased diode, while the forward biased one plays negligible role due to its low resistance ^64^. In dark mode, the holes in the CGO cannot go through the Schottky barrier and only a small current (reverse saturation current) is generated by drift of carriers in the presence of the electric field. By applying light illumination, electron–hole carriers are produced in the depletion region, which pass through the reversed bias diode by thermionic emission mechanism. In fact, this current is the reverse saturation current, which increases linearly with the photon flux in the case of light irradiation. Thus, the I–V curve for Cu/CGO/Cu structure in the negative applied voltage branch is similar to that of a conventional reverse biased diode under irradiation. In the positive applied voltage branch, however, the other diode is in the reverse bias mode and the previous diode is in the forward bias. The behavior of the system is repeated once again with altered current polarization and the symmetrical shape of this two back-to-back diode system is justified.

The photoconductivity is due to the excess charge carriers which are formed by irradiating light to the semiconductor. In this way, photo-detection is accomplished by increasing light-induced excess charge carriers. However, surface related processes can also be effective in the photoconductivity mechanism ^65^. Surface effects become substantial in photodetectors based on n-type ZnO nanostructures^25^. O_2_ molecules are adsorbed at the surface of the nanostructures in the dark mode by the reaction:

4 $$O_{2} \left( {gas} \right) + e^{ - } \to O_{2 }^{ - } \left( {ad.} \right)$$

During this reaction, O_2_ molecules capture electrons from the conduction band of ZnO. In this way, electron carriers are reduced and a depletion region as well as a potential barrier are stablished, which reduces the dark current. Subsequently, by light radiation and the generation of electron–hole pairs in ZnO, some holes move towards the surface and during a recombination process, they release oxygen ions from the surface:

5 $$h^{ + } + O_{2 }^{ - } \left( {ad.} \right) \to O_{2} \left( {gas} \right)$$

As a result of this surface desorption, the captured electrons are released and return to the conduction band of ZnO, which increases the conductivity^66^. The oxygen adsorption/desorption process on the surface causes decline in the dark current and growth in the photocurrent, which eventually benefits to improve the photoconductivity performance of the n-type ZnO semiconductor. This mechanism is different in p-type semiconductors. For example, it has a distinct effect on photoconductivity and even causes negative photoconductivity in p-type ZnSe nanostructures^67^. ${O}_{2 }^{-}$ ions are formed on ZnSe surface according to the Eq. (4) by oxygen adsorption in dark mode. Capturing an electron in this p-type material is identical to releasing a hole in the valence band. By increasing the hole concentration in dark mode, the conductivity rises and the dark current increases. By light illumination and generating electron–hole pairs, the holes can release adsorbed oxygen, as before. This indicates the holes, which were released before, are recombined and annihilated. So, the conductivity of this p-type material decreases under light irradiation by reducing hole concentration, which causes negative photoconductivity. Still, the severity of these effects is highly dependent on the effective surface of the semiconductor. The oxygen adsorption/desorption process can be considered dominant in nanostructures where the surface area/volume ratio is very high. However, in thin-films that possess compact structure, the surface area/volume ratio is considerably low. The density of atoms on the surface and in the bulk can be considered approximately 10^15^ cm^−2^ and 10^23^ cm^−3^, respectively^68^. Regarding the CGO thin-films, as FESEM images also show, it can be assumed that the effective surface that is in contact with the atmosphere is much lower than what we perceive in nanostructures. In a porous ZnO semiconductor, the specific surface area (SSA) can be estimated even to values greater than 100 m^2^/g^69^. This specific surface area will be thousands of times smaller for dense thin films, so that, the oxygen adsorption/desorption process can be almost ignored. According to the mechanism of oxygen adsorption/desorption in p-type materials, it can be imagined that negative photoconductivity should be seen in CGO in case of dominant surface related processes. However, such observations are not seen in our experimental results. Oxygen adsorption/desorption may reduce a portion of photocurrent in CGO, but the share of these effects is so small that its presence is not realized. In this way, the dominant mechanism of photoconductivity can be attributed to the generation of electron–hole pairs in the CGO bulk and their transport by the electric field.

One can estimates the responsivity ($R$) of the devices according to the relationship^70^;

6 $$R = \left( {I_{Ph} - I_{dark} } \right)/P_{in}$$

In this equation, ${I}_{Ph}$, ${I}_{dark}$, and ${P}_{in}$ are the current under illumination (photocurrent), dark current, and incident ligth power, respectively. Responsivity is a benchmark to evaluate the performance of the PDs. The responsivity of Ag, Cu and Ni samples is 85, 29, and 3 mA/W at − 5 V bias voltage, respectively. Considering the Ohmic nature of Ag contact and the formation of photoconductor-type device, it can be seen that the responsivity in this sample is much higher than that of the other samples. As previous studies also confirmed, the responsivity of photoconductor devices is generally higher than that of the photodiodes and systems based on Schottky contacts^71^. Superior responsivity of the Ag sample compared to the Ni sample can be due to the lower specific resistance of silver. The formation of an Ohmic contact is not only related to the location of the energy levels, but also depends on contact resistance. As stated by definition, an Ohmic contact is defined as a metal/semiconductor junction that has a negligible resistance relative to the total resistance of the semiconductor device^72^.

The dark current (at − 5 V bias) in Ag, Cu, and Ni samples is 670, 1.36, and 1710 nA, respectively. The dark current in the Cu sample is very low, which is the Schottky systems’ characteristic. The Schottky barrier causes inferior charge flow from the metal into the semiconductor, in the dark mode. Low dark current is considered as a decisive factor in PDs. This quantity can be expressed more intelligently in the form of the noise equivalent power (NEP) or specific detectivity (${D}^{*}$) which has inverse relationship with square root of the dark current^73^:

7 $$D^{*} = R\sqrt {\frac{A}{{2qI_{dark} }}}$$

In this equation $A$ is the active area of the PD and $q$ denotes the electron charge. Specific detectivity describes how sensitive is the PD to a weak signal. ${D}^{*}$ for Ag, Cu, and Ni samples is 6.37 × 10^10^, 4.72 × 10^11^, and 1.49 × 10^9^ Jones, respectively. The Cu-based PD, which is a Schottky-MSM, has highest ${D}^{*}$ and is more suitable for sensitive detections. Moreover, the photo- to dark current ratio (PDCR) in − 5 V bias voltage and 0.15 mW incident power for Ag, Cu, and Ni samples is 20, 3144, and 2.5. The higher value for this ratio in the Cu sample also indicates the greater sensitivity of this device to incident light.

Figures 7d, e determine the I–T curves of Ag and Cu samples in consecutive ON and OFF lighting modes in the same period intervals. The photocurrent rises by increasing the incident power, which has a good match with the I–V curves. As can be seen, in 60 s periods, when the light source is turned ON, the current reaches its saturation state. These figures indicate how reliable the PDs are and how well the results are repeated at different incident powers. The long-term stability of the fabricated photodetectors was also investigated. For this purpose, the stability of Ag and Cu photodetectors was examined over 20 consecutive ON/OFF cycles. The results of this test can be seen in Figure S2 of the supplementary information. The photocurrent of each device is reproduced in each period, which shows the reproductivity of the devices and their long-term stability. However, the photocurrent in the first two periods is not completely established, which can be due to that the system has not reached to thermal equilibrium in the initial moments.

The Fig. 7f plots the transient behavior of three samples of Ag, Cu, and Ni in one period time of I–T curves. The LED source is quickly turned ON and the photocurrent is measured by the source-meter until the current reaches the operating point current of the system and becomes saturated. The LED is then turned OFF at once to measure the current decay as a function of time. The PD’s response time is measured by 10 to 90% of the maximum current^74^. The response time is termed rise time and decay time when the system is turned ON and OFF, respectively. The rise time for Ag, Cu, and Ni samples is 12.2, 1.8, and 23.1 s, respectively, and the decay time for corresponding samples is 12.8, 5.9, and 25.7 s. As can be seen, the rise and decay time for the Cu sample is much shorter than that of the Ag and Ni samples. The fact that the Schottky-based PDs are faster than Ohmic ones has been realized many times in literature^75–78^. This can be explained as follows. In the Schottky junction, there is always a Schottky barrier that causes energy bands to bend. This band bending renders the presence of a strong electric field or a depletion region. In this case, with the light absorption and generation of electron–hole pairs, the separation of electron–hole in this region is done quickly, which increases the response speed of the device. However, the responsivity of the Cu sample is inferior to that of Ag. This observation has also been reported in most of the studies^79^. In actual fact, there is always a trade-off between responsivity and response time; generally, increasing responsivity leads to longer response time and vice versa^80,81^. In addition to the S900 sample, photodetectors based on the other layers synthesized at temperatures of 600, 700 and 800 °C were also prepared using Ag contact. The I–V curves of these devices can be seen in Figure S3 under UV illumination (0.15 mW incident power). The photocurrent for S600, S700, and S800 samples are lower than that of the S900 sample, which can be attributed to the multiphase structures and lower conductivity in these layers. The dark current is almost the same for all the samples due to the silver electrode. Therefore, reducing the photocurrent in the samples apart from S900 means reducing the responsivity. The reduction of responsivity in these samples leads to inferior specific detectivity. Furthermore, the transient response curves of S600, S700, and S800 photodetectors in Figure S3 show that S600 and S700 samples have a longer response time, which can be caused by defects in these semiconductors. By intensification of defects, more electrons and holes are trapped in the defect levels, which delays the extraction and collection for charge carriers, so, the response time upsurges. It can be concluded that choosing the S900 sample for constructing the photodetectors was a sensible choice.

In the last two decades, several attempts have been made to fabricate CGO-based PDs. However, research has not been extensively conducted on this material for PD application. Li et al.^82^, fabricated (CGO nanoplate)/(ZnS microsphere) UV-PD, but did not achieve any photo response. Wang et al.^13^ also fabricated nanostructure-based CGO PD and reached a responsivity of 0.033 A/W by 365 nm illumination at 10 V bias voltage. Tsay and Chen^41^ also obtained a responsivity of 0.08 A/W by manufacturing MSM-type thin film CGO PD. Shi et al.^36^ designed a deep-UV-PD based on CuGaO_2_/β–Ga_2_O_3_ heterojunction. There is no responsivity reported for this self-powered PD, which works at zero bias. The response time and PDCR are reported to be less than 500 ms and more than 40, respectively. Wang et al.^2^ also fabricated CGO NP-based and CGO NP:Cr/ZnO PDs, for which responsivities of 0.09 and 31 mA/W were achieved, respectively. Li et al.^31^ attained responsivities of 0.64 and 1.34 A/W by establishing CGO:Mg/ZnO and CGO:Zn/ZnO heterostructures, respectively. All the CGO-based PDs that have been investigated to date are listed in Table 3 along with their characteristic parameters.

**Table 3:** Specifications for fabricated PDs and comparison with other CGO-based UV-PDs. NS and HJ denote nanostructure and heterojunction, respectively.

Sample	Structure	I_dark_ (nA)/Bias (V)	Responsivity (mA/W)/Wavelength (nm)	Detectivity (Jones)	Rise/Decay time (s)	Photo to dark current ratio (PDCR)	Ref
CGO/ZnS	NS-HJ	3.5 × 10^–3^/5	− /280–400	–	–/–	1.6	^82^
CGO NPs	NS	9.95 × 10^+5^/10	33/365	–	0.7/0.3	0.19	^13^
CGO:Zn NP/ZnO NW	NS-HJ	1.5 × 10^+5^/10	120/365	–	0.8/0.4	4.5	^13^
Au/CGO/Au	Thin film-MSM	2.76 × 10^+5^/ −	80/UVC	–	~ 48/ ~ 59	1.02	^41^
CGO/β-Ga_2_O_3_	Thin film-HJ	− /0	− /254, 365	–	< 0.5/ < 0.5	> 40	^36^
CGO:Cr NPs	NS	− /10	0.09/365	–	–/–	2	^2^
CGO:Cr/ZnO/Ag	NS-HJ	− /10	31/365	–	2.95/3.29	10	^2^
CGO:Mg/ZnO	NS-HJ	− /10	636/365	–	–/–	1.74	^31^
CGO:Zn/ZnO	NS-HJ	− /10	1340/365	–	–/–	6.03	^31^
Ni/S900/Ni	Thin film-MSM	1.71 × 10^+3^/5	3/365	1.49 × 10^9^	23.1/25.7	2.5	This work
Cu/S900/Cu	Thin film-MSM	1.36/5	29/365	4.72 × 10^11^	1.8/5.9	3144	This work
Ag/S900/Ag	Thin film-MSM	670/5	85/365	6.37 × 10^10^	12.2/12.8	20	This work

The dependence of $R$, ${D}^{*}$, and ${I}_{Ph}$ on the incident power is part of the device's performance, which can be seen in Fig. 7g. Although, the photocurrent increases with the incident power, the responsivity and specific detectivity decrease exponentially for all the samples. The responsivity decay can be explained by Eq. (6). ${I}_{Ph}$ increases with ${P}_{in}$, but this photocurrent increasement is not the only determining factor to rise $R$, so as ${P}_{in}$ increases in the denominator, $R$ decreases. For example, by 3.2 times increasement of the incident power from 0.15 to 0.48 mW, the photocurrent has grown only 1.36 times for the Ag sample. This means that the current growth cannot compensate the power increment. The photocurrent growth becomes less and less at higher incident powers so that a constant current will be established at very high powers (because the material cannot produce more electron–hole pairs), and therefore the responsivity tends to zero. On the contrary, at low incident powers, even a very dim light can generate a lot of electron–hole pairs and produce substantial photocurrent compared to the dark current. Consequently, by lowering the incident power, the responsivity growth rapidly. In this sense, Schottky-based PDs are crucial devices in detecting weak optical signals. Referring to Eq. (7), the same argument can be practical for ${D}^{*}$. Excluding $R$, the rest of the parameters are constant, so, ${D}^{*}$ reduces in the same way as the power rises.

A photoconductive detector operates by registering the photocurrent, which is proportional to the incident photon flux^83^. Therefore, as long as the power-current relationship is linear, the performance of the PD is reliable. An ideal PD shows a linear I–P characteristic in its dynamic range. However, practical PDs do not always have a linear I–P characteristic and only behave linearly within a definite range. The non-ideal performance of a device can be expressed by the power law equation ^84^;

8 $$I = AP^{\theta }$$

where $A$ is a constant and the power factor $\theta$ is an experimental constant, which can be estimated by fitting the I–P curve (generally $0.5<\theta <1$). By an allometric curve fitting in Fig. 7g, $\theta$ is calculated as 0.68, 0.71, and 0.65 for Ag, Cu, and Ni samples, respectively. Tending $\theta$ to 1 means an ideal device. It is believed that $\theta$ is related to the trap states or defects in the semiconductor^85^. Considering the same semiconductor used in all three samples (S900), the closeness of $\theta$ values is conceivable.

The CGO-based PD can be prepared with the planar MSM structure by the same electrodes of Ag, Cu, or even Ni. However, the devices based on nickel contact do not have very promising results. The device with silver contact will have a long response time even though it shows a good responsivity. In contrast, the device based on copper electrode, although is not very responsive, it performs faster. This behavior is similar to the trend that we had mentioned in our previous article^22^ only by simulating the CGO PDs. From application point of view, it is possible to prepare a specific electrode for the MSM structure and utilize it in different situations.

## Conclusions

In this study, we synthesized α-CGO thin films via a facile method. The thin films included various phases of CuO, spinel and delafossite after heat treatment at different temperatures, but the pure delafossite phase was attained at 900 °C annealing temperature. We realized that the presence of different phases causes grain boundaries and decrease in the conductivity of the material. The optical investigations showed that by increasing temperature up to 900 °C, the optical and structural characteristics of the layers have gradually improved. The hole concentration and conductivity of the annealed thin films at 900 °C are 8.98 × 10^16^ cm^−3^ and 2.24 × 10^−3^ S/cm respectively, which indicate high electronic properties. Moreover, we investigated the contact material influence by fabricating MSM-type UV-PDs. The performance of the PD can be altered by choosing various contacts. The PD based on the silver contact presents a good photo response with Ohmic characteristics, while the Cu contact shows an increase in the speed of the PD benefiting Schottky properties.

## Supplementary Information

Supplementary Information.

## Data Availability

The datasets used and/or analyzed during the current study available from the corresponding author on reasonable request.
