# Supplementary material for: Investigating various metal contacts for p-type delafossite α-CuGaO2 to fabricate ultraviolet photodetector
Source: Sci Rep. 2023 May 22;13:8259. doi: 10.1038/s41598-023-35458-0 (PMC10202953; doi:10.1038/s41598-023-35458-0)
Supplement: Supplementary file 1 — Supplementary Information. [file 41598_2023_35458_MOESM1_ESM.docx]

Supplementary Information

Investigating Various Metal Contacts for p-type Delafossite α-CuGaO_2_ to Fabricate Ultraviolet Photodetector

Masoud Abrari ^a^, Majid Ghanaatshoar ^a^, Shahab Sharifi Malvajerdi ^b^, Saeb Gholamhosseini ^a^, Alireza Hosseini ^a^, Haiding Sun ^b^, Seyed Majid Mohseni ^c^

^a^ Laser and Plasma Research Institute, Shahid Beheshti University, 1983969411 Tehran, Iran;

^b^ School of Microelectronics, University of Science and Technology of China, Hefei, Anhui 230026, China;

^c^ Department of Physics, Shahid Beheshti University, 1983969411 Tehran, Iran;

Corresponding authors:

Majid Ghanaatshoar: [m-ghanaat@sbu.ac.ir](mailto:m-ghanaat@sbu.ac.ir)

Shahab Sharifi Malvajerdi: [sharifimalvajerdi@mail.ustc.edu.cn](mailto:sharifimalvajerdi@mail.ustc.edu.cn)


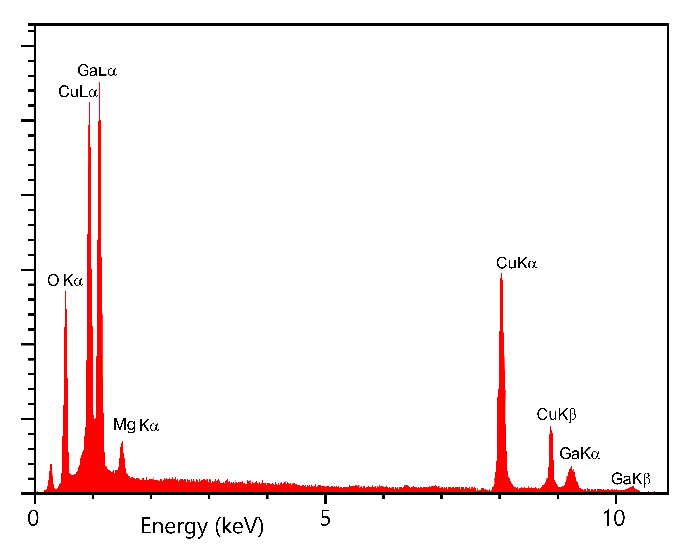


**Atomic ratio (%)**

**Cu Ga O Mg**

**20 24 54 2**


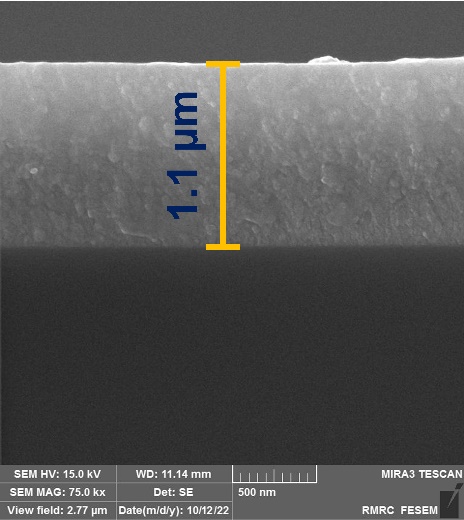


Figure S1: Cross-sectional FESEM image and EDS of S900 layer with more than 1 µm thickness.

Figure S2: Long-term stability of the photodetectors based on Ag and Cu contacts over 20 consecutive ON/OFF cycles.

Figure S3: I-V curves and transient response for photodetectors based on S600, S700, S800 and S900 samples with Ag contact.
